# Association between the triglyceride glucose index and diabetic retinopathy in type 2 diabetes: a meta-analysis

**DOI:** 10.3389/fendo.2023.1302127

**Published:** 2023-12-07

**Authors:** Jianlong Zhou, Lv Zhu, Yadi Li

**Affiliations:** ^1^ Department of Traditional Chinese Medicine, People’s Hospital of Deyang City, Deyang, China; ^2^ Department of Integrative Medicine, West China Hospital, Sichuan University, Chengdu, China

**Keywords:** triglyceride glucose index, diabetic retinopathy, type 2 diabetes, meta-analysis, observational study

## Abstract

The triglyceride-glucose (TyG) index is an accessible and reliable surrogate indicator of insulin resistance and is strongly associated with diabetes. However, its relationship with diabetic retinopathy (DR) remains controversial. This meta-analysis aimed to assess the relationship between the TyG index and the prevalence of DR. Initial studies were searched from PubMed, Embase, Web of Science, and China National Knowledge Infrastructure (CNKI) electronic databases. The retrieval time range was from the establishment of the database to June 2023. Pooled estimates were derived using a random-effects model and reported as odds ratio (OR) with 95% confidence intervals (CIs). Two researchers independently assessed the methodological quality of the included studies. The Newcastle-Ottawa Quality Scale (NOS) was utilized to assess cohort studies or case-control studies. The Agency for Healthcare Research and Quality (AHRQ) methodology checklist was applied to assess cross-sectional studies. Ten observational studies encompassing 13716 patients with type 2 diabetes were included in the meta-analysis. The results showed that a higher TyG index increased the risk of DR compared with a low TyG index (OR: 2.34, 95% CI: 1.31-4.19, P < 0.05). When the index was analyzed as a continuous variable, consistent results were observed (OR: 1.48, 95% CI: 1.12-1.97, P < 0.005). There was no significant effect on the results of the sensitivity analyses excluding one study at a time (P all < 0.05). A higher TyG index may be associated with an increased prevalence of DR in patients with type 2 diabetes. However, high-quality cohort or case-control studies are needed to further substantiate this evidence.

**Systematic review registration:**

https://www.crd.york.ac.uk/PROSPERO/, identifier CRD42023432747.

## Introduction

Diabetic retinopathy (DR) is a common and serious chronic microvascular complication of diabetes mellitus (DM) (1). It is one of the leading causes of vision loss worldwide (2), as well as the leading cause of vision impairment in patients aged 25-74 (3). A recent meta-analysis of 59 population-based studies has shown that the global prevalence of DR is 22.27% and the prevalence of vision-threatening diabetic retinopathy (VTDR) is 6.17% among people with DM. In 2020, the global number of adults with DR and VTDR was estimated to be 103.12 million and 28.54 million, respectively; by 2045, it is expected to increase to 160.5 million and 44.82 million, respectively (4). DR not only affects patients’ visual quality, but it is also associated with patients’ health-related quality of life and well-being. As DR progresses, the patient’s ability to work and live continues to decline, while some DR patients also experience varying degrees of anxiety or depression (5, 6). Therefore, early diagnosis and prevention are important to reduce the prevalence of DR and the hazard caused by adverse prognosis.

Insulin resistance (IR), referred to as resistance to insulin-stimulated glucose uptake, is an essential feature of aging, vascular disease, obesity, type 2 diabetes mellitus (T2D), dyslipidemia, and other components of the metabolic syndrome (7, 8). It has been found that IR may be an early driver of DR in the absence of significant hyperglycemia (9). The hyperinsulinemic-euglycemic clamp is a gold-standard method for assessing IR, but it is cumbersome, expensive, and not easy to perform in the clinic (10). Interestingly, the triglyceride-glucose (TyG) index, an indicator calculated from fasting triglyceride (TG) and fasting plasma glucose (FPG), is a simple, accurate, and reliable surrogate marker for assessing IR (11).

Previous studies have confirmed that the TyG index was strongly associated with DM (12), diabetic nephropathy (DN) (13), metabolic syndrome (MetS) (14), and atherosclerotic cardiovascular diseases (ASCVDs) (15). Recent studies have shown that the TyG index is a favorable predictor of DR prevalence and incidence (16, 17). Nevertheless, some studies have shown no correlation between the TyG index and DR (18, 19). The different results may be due to differences in factors such as participants, inclusion criteria, and type of study design. However, it still showed that the relationship between the TyG index and DR is controversial. Therefore, we performed this systematic review and meta-analysis aimed at assessing the relationship between the TyG index and DR.

## Methods

This study followed the Preferred Reporting Items for Systematic Evaluation and Meta-Analysis (PRISMA) guidelines for meta-analysis. A detailed PRISMA checklist has been provided in supplementary material 1. The study was registered on the PROSPERO platform (CRD42023432747).

### Sources and methods of data retrieval

The literature search used the following combination of words: (1) “triglyceride glucose index” OR “TyG index” OR “triglyceride and glucose index” OR “triglyceride–glucose index” OR “triglyceride/glucose index” OR “triacylglycerol glucose index;” and (2) “diabetic retinopathy” OR “diabetic retinopathies” OR “diabetes retinopathy” OR “retinopathy, diabetic”. The following electronic literature databases will be searched: PubMed, Embase, Web of Science, and CNKI. Literature published in English or Chinese will be included. It was limited to human studies only. The search strategy will be conducted in duplicate and independently. The detailed search strategy for PubMed is described in supplementary material 2, and similar search strategies will be used for other electronic databases. Prior to the final analysis, the search will be re-run periodically until June 2023 to retrieve additional eligible studies, excluding unpublished studies.

### Study selection and eligible criteria

Studies meeting the following criteria will be included: (1) the research design was observational study; (2) study participants were clearly diagnosed with T2D; (3) the TyG index could be acquired by laboratory examination; (4) DR was definitively diagnosed as the outcome disease; (5) the outcome measures indicating the relationship between TyG index and DR risk were presented as odds ratios (ORs), risk ratios(RRs), or hazard ratios (HRs), along with their corresponding 95% confidence intervals (CIs). Alternatively, complete data that could be used to calculate the effect size was provided; and (6) the effect estimates were obtained after accounting for confounding variables.

Meanwhile, studies meeting the following criteria will be excluded: (1) conference abstracts; (2) *in vitro* or animal experiments; (3) duplicate literature; (4) editorials, reviews, or commentaries; and (5) studies lacking sufficient data.

### Date extraction and quality assessment

Two researchers independently conducted a literature search and screening. Subsequently, they performed data extraction and quality assessment of the eligible literature. In case of disagreement, it would be resolved by the third researcher. Based on the included studies, we extracted the following data: first author, year of publication, country of subjects, research design, number of participants, demographics (age, sex), model for TyG index analysis, and confounding variables adjusted in multiple regression analyses. The TyG index was calculated according to the following formula: ln (fasting TG [mg/dL] × fasting glucose [mg/dL]) (20). The estimates of effect were recorded as odds ratios (ORs). OR was defined as odds ratios, RRs, or HRs.

Depending on the type of observational study, the two authors chose different assessment tools to independently assess the quality and risk of bias of the included studies. The Newcastle-Ottawa Scale (NOS) was used to assess case-control studies and cohort studies (21). For cross-sectional studies, the Agency for Healthcare Research and Quality (AHRQ) methodology checklist was selected for assessment (22). The NOS consisted of eight items categorized into three aspects such as selection, comparability, and outcome or exposure. Based on the number of stars assessed, the quality of the literature was differentiated into low quality (< 5 stars), medium quality (5-7 stars), and high quality (> 7 stars). AHRQ’s assessment methodology included the eleven-item assessment component. The quality of the literature was ranked according to the percentage of “yes” received as low quality (< 30%), medium quality (30%-60%), and high quality (> 60%).

### Statistical analysis

Statistical analysis was performed using Revman version 5.4, STATA version 12.0 and R version 4.4.1 softwares. For categorical and continuous data, we summarized the OR and the corresponding 95% CI to assess the relationship between the TyG index and the prevalence of DR, respectively. The effect valuation was seen to be statistically significant when the *P*-value was < 0.05. If the TyG index was treated as a categorical variable, we would extract the estimation value from the highest TyG index level. Once the TyG index was assessed as a continuous variable, we would extract the estimation value for each unit increase in the variable. If multiple models were present in the multifactorial analysis, we chose the one with the most adjusted confounders. To evaluate heterogeneity, Cochrane *Q* and *I^2^* tests were employed (23). When the *P*-value of the *Q*-test was less than 0.1 or *I^2^* > 50%, it indicated the existence of significant heterogeneity. If *I^2^* > 75%, it signified the presence of a high degree of heterogeneity. If there was significant heterogeneity, a random effects model was taken. Conversely, a fixed-effects model was employed. To test the stability of the findings, we further undertook sensitivity analyses, in which one study was excluded at a time and the change in the pooled OR of the remaining studies was observed (24).

To assess potential publication bias, we adopted funnel plots and contour-enhanced funnel plots incorporating the “cut-and-fill method” (25). Besides, we also used Egger’s test to assess publication bias. If the funnel plot existed asymmetrically and the missing studies were located in regions with no statistical significance, the funnel plot asymmetry may be caused by publication bias. If publication bias was present, the pooled risk estimates were recalculated using the cut-and-fill method. If the funnel plot was asymmetric and the missing studies were distributed in statistically significant areas, this showed that the funnel plot asymmetry could be caused by other reasons rather than publication bias.

### Patient and public involvement

Patients and the public were not involved in this study.

## Results

### Literature search

**Figure 1:**
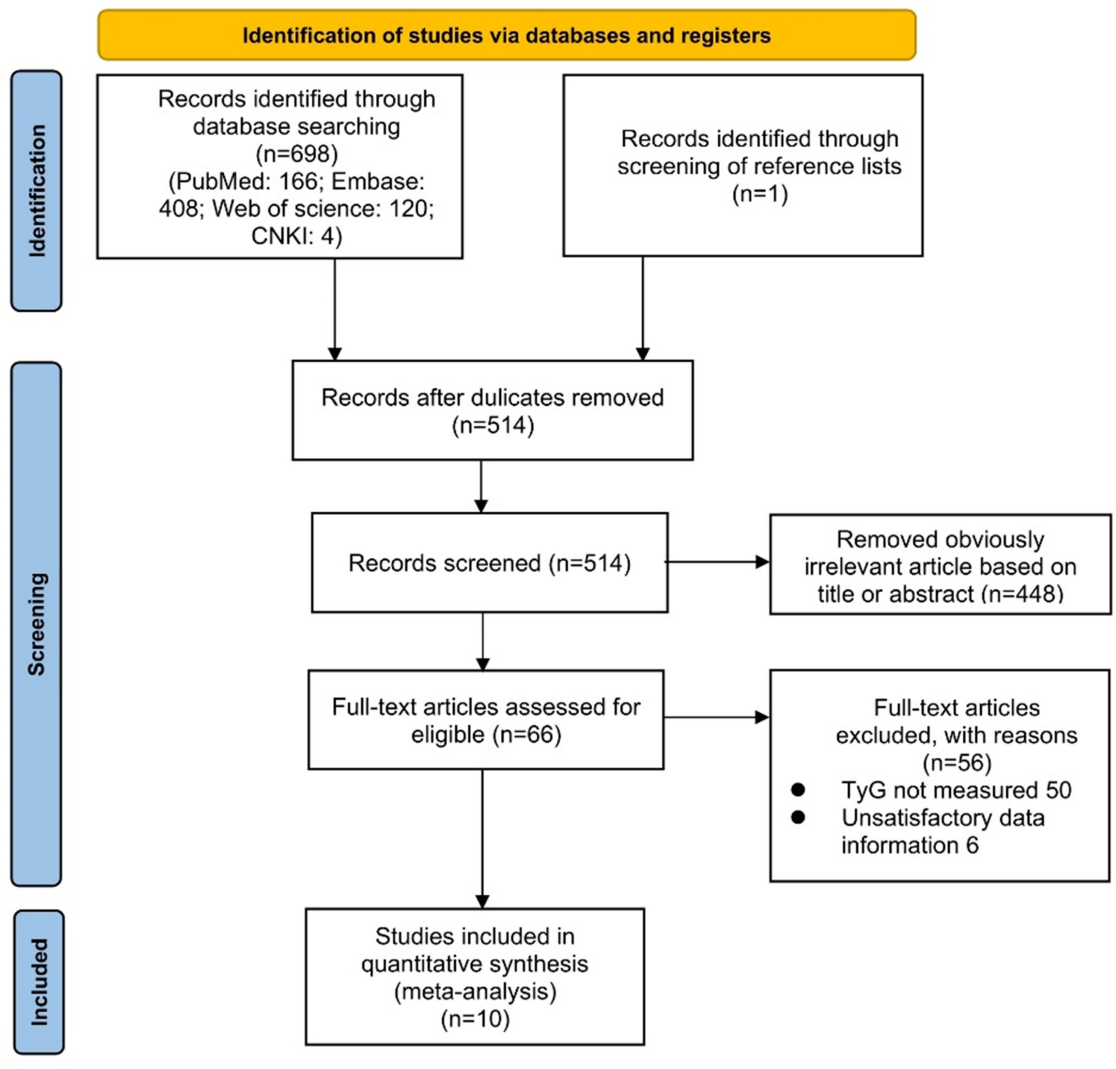
PRISMA flow diagram of literature search.

According to the search strategy, we retrieved a total of 699 literatures. Potentially relevant 514 pieces of literature were retained after the exclusion of duplicates. Then, 448 pieces of literature that were obviously irrelevant were excluded based on their titles or abstracts. Afterward, we assessed the remaining 66 pieces of literature across the full text. Eventually, ten qualified studies were integrated into the meta-analysis. The procedure for a more detailed literature search was illustrated in **Figure 1**.

### Characteristics

In the present meta-analysis, we incorporated ten observational studies that encompassed a substantial sample size of 13,716 individuals in the quantitative synthesis. Among them were three cohorts studies (16, 19, 26), one case-control study (17), and six cross-sectional studies (27–32). These studies were conducted in four different countries such as Iran, Singapore, China, and India. The study primarily involved adults diagnosed with type 2 diabetes, although one study did not provide specific details about the type of diabetes under consideration (29). Nevertheless, considering the mean age of the included patients, it is reasonable to assume that they were type 2 diabetics. The number of participants ranged from 208 to 4721. Furthermore, the mean age of all participants across the studies exceeded 50 years. The percentage of male participants varied from 46.63% to 86.47%. The TyG index was used as the categorical variable in four studies and as the continuous variable in three studies. Another three studies utilized the TyG index as both types of variables. The confounders adjusted were nonidentical among the different studies. Adjusted confounders usually encompassed age, gender, duration of diabetes, body mass index (BMI), glycosylated hemoglobin, blood pressure, high-density lipoprotein (HDL), low-density lipoprotein (LDL), and other factors. However, in one study, adjusted confounders were not clearly identified (27). The information on the baseline characteristics of the included studies was presented graphically in **Table 1**.

**Table 1:** Characteristics of the included studies.

Study	Country	Research type	Characteristics of participants	Number of participants	Mean age (years)	Male (%)	TyG index analysis	Variables adjusted
Hameed EK, et al., 2019 (27)	Iraq	Cross-sectional	T2DM patients	416	NDR: 54.56 ± 9.31; DR: 58.98 ± 7.63	46.63%	Q4: Q1	Age, duration of diabetes, FBG, HbA1C, SBP, DBP, TC, TG, HDL, WC, and BMI
Neelam K, et al., 2023 (16)	Singapore	Cohort	T2DM patients	1339	NDR: 56.1 ± 10.7; DR: 56.5 ± 9.4	55.90%	Continuous	Duration of type 2 diabetes, BMI, eGFR, uACR and SBP
Pan Y, et al., 2021 (19)	China	Cohort	T2DM patients	4721	59.56 ± 13.02	53.57%	Continuous	Age, sex, BMI, smoking status
Srinivasan S, et al., 2021 (28)	India	Cross-sectional	T2DM patients	1413	56.30 ± 10	53.01%	Continuous; Categorized	Age, smoking, blood pressure
Yao LT, et al., 2021 (17)	China	Case-control	T2DM patients	2112	56.08 ± 13.85	57.90%	Q4: Q1	Age, sex, duration of diabetes, use of antidiabetic agents, HR, SBP, PP, height, weight, BMI, HbA1c, and TC
Zhou Y, et al., 2023 (29)	China	Cross-sectional	Adults with diabetes mellitus	888	62.2 ± 12.1	50.11%	Continuous; Categorized	Age; gender; race; education; PIR; HDL; LDL; TC; hypertension and retinopathy
Wang J, et al., 2022 (30)	China	Cross-sectional	T2DM patients	1061	NDR: 60.07 ± 8.06; DR: 57.63 ± 8.45	82.09%	Q4: Q1	Gender, age, smoking history, the course of diabetes, HbA1c, SBP, DBP, BMI, and SUA
Pang M, et al., 2020 (31)	China	Cross-sectional	T2DM patients	208	NDR: 53.68 ± 14.39; DR: 54.85 ± 11.37	61.06%	Continuous	Duration of diabetes, SBP, SUA
Li CH, et al., 2022 (26)	China	Cohort	T2DM patients	1153	58.89 ± 8.60	86.47%	Q4: Q1	Age, gender, course of disease, smoking, alcohol consumption, exercise, HDL-C, SBP, BMI, HbA1c, the use of hypoglycemic drugs, and the use of lipid-lowering drugs
Xiao HY, et al., 2022 (32)	China	Cross-sectional	T2DM patients	405	58.9 ± 9.7	56.50%	Continuous; Categorized	Gender, age, Duration of type 2 diabetes, BMI

NDR, no diabetic retinopathy; DR, diabetic retinopathy; FBG, fasting blood glucose; BMI, body mass index; eGFR, estimated glomerular filtration rate; uACR, urinary albumin-to-creatinine ratio; SBP, systolic blood pressure; DBP, diastolic blood pressure; HR, heart rate; PP, pulse pressure; HbA1c, glycated hemoglobin; TC, total cholesterol; PIR, poverty income ratio; HDL, high-density lipoprotein; HDL-C, HDL-cholesterol; LDL, low-density lipoprotein; WC, waist circumference; SUA, serum uric acid.

### Quality assessment

The AHRQ methodology checklist was used for quality assessment of the six cross-sectional studies, as shown in **Table 2**. Of these, three studies were evaluated as high quality. Three studies were assessed as medium quality. The NOS was used to assess the literature quality of one case-control study and three cohort studies, as shown in **Table 3**. There were two studies evaluated as high quality and two studies evaluated as medium quality. Overall, the studies included in this meta-analysis demonstrated a relatively good level of quality.

**Table 2:** Quality Assessment of cross-sectional studies with AHRQ methodology checklist.

Domain	Hameed EK, et al., 2019 (27)	Srinivasan S, et al., 2021 (28)	Zhou Y, et al., 2023 (29)	Wang J, et al., 2022 (30)	Pang M, et al., 2020 (31)	Xiao HY, et al., 2022 (32)
1 Define source of information (survey, record review)	Y	Y	Y	Y	Y	Y
2 List inclusion and exclusion criteria for exposed and unexposed subjects (cases and controls) or refer to previous publications	Y	Y	Y	Y	Y	Y
3 Indicate time period used for identifying patients	Y	N	Y	Y	Y	Y
4 Indicate whether or not subjects were consecutive if not population-based	Y	N	U	U	Y	U
5 Indicate if evaluators of subjective components of study were masked to other aspects of the status of the participants	N	N	N	N	N	N
6 Describe any assessments undertaken for quality assurance purposes (e.g., test/retest of primary outcome measurements)	Y	Y	Y	Y	Y	Y
7 Explain any patient exclusions from analysis	Y	U	Y	Y	N	N
8 Describe how confounding was assessed and/or controlled	Y	Y	Y	Y	Y	Y
9 If applicable, explain how missing data were handled in the analysis	U	U	U	U	U	U
10 Summarize patient response rates and completeness of data collection	U	N	Y	Y	U	N
11 Clarify what follow-up, if any, was expected and the percentage of patients for which incomplete data or follow-up was obtained	N	N	N	N	N	N
Number (percentage) of domain agreement	7/11 (64%)	4/11 (36%)	7/11 (64%)	7/11 (64%)	6/11 (55%)	5/11 (45%)

AHRQ, Agency for Healthcare Research and Quality; Y, Yes; N, No; U, Unclear.

**Table 3:** Quality assessment of the Newcastle-Ottawa Scale for cohort and case-control studies.

Author (year)	Selection	Comparability	Outcome/Exposure	NOS score
Neelam K, et al., 2023 (16)	★★★	★★	★★★	8
Pan Y, et al., 2021 (19)	★★★	★★	★★	7
Li CH, et al., 2022 (26)	★★★	★★	★★★	8
Yao LT, et al., 2021 (17)	★★★	★★	★★	7

★: The NOS consists of eight items categorized into three aspects. Each numbered item can score one star if the study is eligible. A maximum of four stars can be awarded for selection, two stars for comparability, and three stars for outcome or exposure.

### TyG index and prevalence of DR

**Figure 2:**
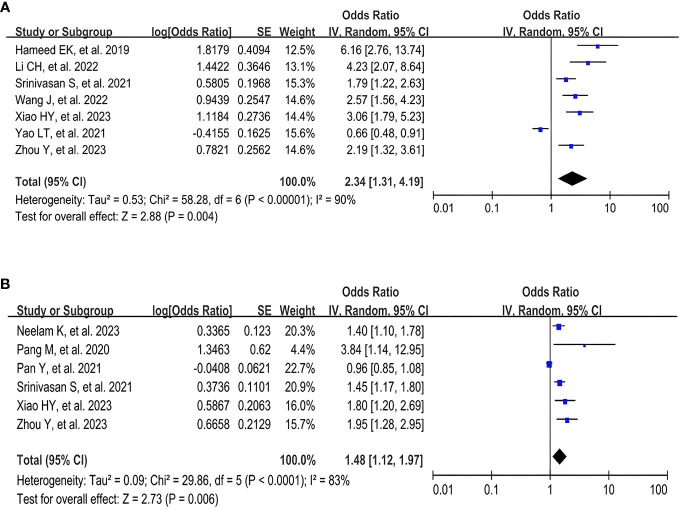
Forest plot of the relationship between TyG index and DR risk. **(A)** Forest plot of TyG index as a categorical variable. **(B)** Forest plot of TyG index as a continuous variable.

A meta-analysis of seven studies (17, 26–30, 32) using the TyG index as a categorical variable showed that the highest TyG index group had a significantly increased risk of DR compared with the lowest TyG index group (OR 2.34, 95% CI 1.31- 4.19, *I* ^2^ 90%, *P* < 0.05) (**Figure 2A**). A pooled analysis of the six studies (16, 19, 28, 29, 31, 32) in which the TyG index was used as a continuous variable also showed consistent results (OR 1.48, 95% CI 1.12- 1.97, *I* ^2^ 83%, *P* < 0.05) (**Figure 2B**). Meanwhile, these analyses also revealed a high degree of heterogeneity among the studies.

### Sensitivity analysis

The results of the sensitivity analysis demonstrated that excluding any of the studies had no notable effect on the pooled OR values (ORs for the TyG index as a categorical variable: 2.04-2.78, all *P* < 0.05; ORs for the TyG index as a continuous variable: 1.40-1.57, all *P* < 0.05) (**Tables 4**, **5**). Interestingly, in the sensitivity analysis of the TyG index as a categorical variable, we found that heterogeneity decreased to a medium degree after excluding Yao LT, et al’s study (17) (*I* ^2^ 53%). While, in the sensitivity analysis of the TyG index as a continuous variable, the heterogeneity became insignificant after excluding Pan Y, et al’s study (19) (*I* ^2^ 17%).

**Table 4:** Results of the sensitivity analysis when the TyG index was applied as a categorical variable.

Dataset excluded	OR	95% CI	*I*² %	*P* for effect
Hameed EK, et al., 2019 (27)	2.04	1.13, 3.65	90%	0.02
Li CH, et al., 2022 (26)	2.14	1.16, 3.96	90%	0.02
Srinivasan S, et al., 2021 (28)	2.49	1.21, 5.14	91%	0.01
Wang J, et al., 2022 (30)	2.32	1.19, 3.61	91%	0.01
Xiao HY, et al., 2022 (32)	2.25	1.18, 4.30	91%	0.01
Yao LT, et al., 2021 (17)	2.78	2.01, 3.85	53%	< 0.00001
Zhou Y, et al., 2023 (29)	2.39	1.21, 4.71	91%	0.01

**Table 5:** Results of the sensitivity analysis when the TyG index was applied as a continuous variable.

Dataset excluded	OR	95% CI	*I*² %	*P* for effect
Neelam K, et al., 2023 (16)	1.54	1.07, 2.20	85%	0.02
Pang M, et al., 2020 (31)	1.42	1.07, 1.87	85%	0.01
Pan Y, et al., 2021 (19)	1.57	1.33, 1.84	17%	< 0.00001
Srinivasan S, et al., 2021 (28)	1.52	1.06, 2.18	84%	0.02
Xiao HY, et al., 2022 (32)	1.43	1.05, 1.94	84%	0.02
Zhou Y, et al., 2023 (29)	1.40	1.04, 1.89	83%	0.02

### Publication bias

**Figure 3:**
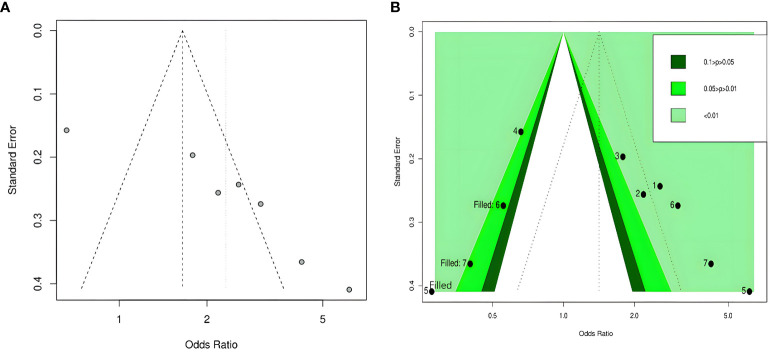
Funnel plot of the relationship between the TyG index as a categorical variable and DR. **(A)** Funnel plot; **(B)** Contour-enhanced funnel plot.

**Figure 4:**
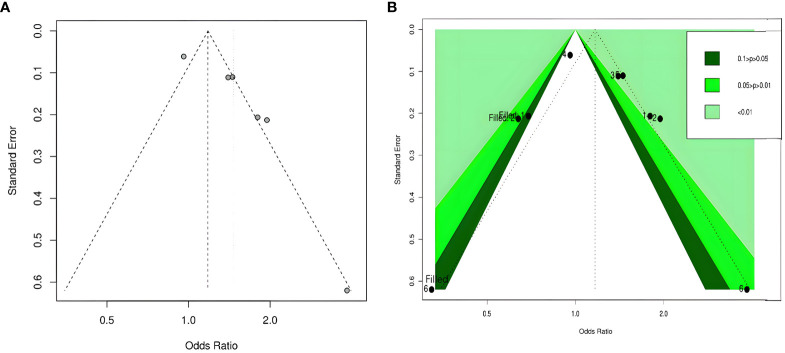
Funnel plot of the relationship between the TyG index as a continuous variable and DR. **(A)** Funnel plot; **(B)** Contour-enhanced funnel plot.

By visual inspection, we found asymmetry in funnel plots (**Figures 3A**, **4A**). Further, we investigated the potential publication bias using a contour-enhanced funnel plot combined with the cut-and-fill method. As shown in **Figures 3B**, **4B**, most of the missing studies were distributed in statistically significant regions, while individual studies (Filled 1 and Filled 6 in **Figure 4B**) were distributed in statistically nonsignificant regions (*P* > 0.05). Therefore, this meta-analysis could not exclude the possibility of publication bias. However, it may also indicate that the observed asymmetry was due to other factors, such as the low number of studies included in the funnel plot analysis, methodological differences in the included studies, and the presence of small sample size studies. Hence, we performed Egger’s test. The results still showed possible publication bias (all *P* < 0.05). However, after recalculating the effect sizes using the cut-and-fill method, there were no statistically significant changes in the effect sizes (adjusted OR for TyG index as a categorical variable: 0.349, 95% CI: -0.208- 0.906, P =0.22, number of cut and fill =3; adjusted OR for TyG index as a continuous variable: 0.158, 95% CI: -0.107- 0.423, P =0.24, number of cut and fill =3), which suggested that publication bias had little effect on the results.

## Discussion

Studies have found that more than 60% of patients with type 2 diabetes might develop diabetic retinopathy within 20 years of its onset (33). As a leading cause of vision loss, DR has a serious impact on the daily life and well-being of patients. Previous studies have found that insulin resistance was a major pathogenic factor in type 2 diabetes (34). Current studies have shown that IR was closely associated with DR (35). However, whether the TyG index, a valid predictor of IR, is associated with DR remains controversial. Our meta-analysis demonstrated that a higher TyG index might be more likely to increase the prevalence risk of DR compared to a lower TyG index. To the best of our knowledge, this study is the first meta-analysis to assess the relationship between TyG index and the risk of diabetic retinopathy in type 2 diabetes.

Risk factors for DR usually involved age, glycosylated hemoglobin (HbA1c), lipids, obesity, duration of diabetes, smoking, kidney disease, hypertension, and others (36, 37). Currently, DR is mainly diagnosed through funduscopic examination (38). According to the Early Treatment Diabetic Retinopathy Research Study (ETDRS) criteria (39), DR was diagnosed if certain characteristic lesions were present, such as cotton wool spots, hard exudates, macular edema, intraretinal microvascular abnormalities, microaneurysms, hemorrhages, or neovascularization. However, there are no obvious symptoms in the early stage of DR (40), and many patients seek medical treatment only when they experience vision loss and blurred vision, which is already a serious condition and is not conducive to later recovery and prognosis. Therefore, it is of great clinical significance to find a convenient and noninvasive early detection index for early screening of DR patients. Our study provided a potential method for early detection of DR. The TyG index could be used to assess the risk profile of DR in patients with type 2 diabetes. Further, optimizing lipid and glycemic management is an important component of diabetes management, which contributes to obtaining the TyG index. Thus, the TyG index, as an accessible routine indicator, may be a potentially clinically valuable option for early diagnosis and treatment of DR.

Although the actual role of the TyG index in the pathogenesis of DR has been unclear, several potential mechanisms associated with IR have been recognized. Previous studies have found that inflammation, oxidative stress, nitric oxide production, mitochondrial damage, and vascular endothelial dysfunction were involved in the pathogenesis of DR (41–45). The increasing evidence suggested that IR played an important role in the mechanism of DR and might be related to the pathways mentioned above (35, 46). C-reactive protein (CRP) is an independent predictor of IR (47). IR may increase the release of inflammatory factors such as C-reactive protein and tumor necrosis factor, leading to the adhesion and aggregation of leukocytes, which can cause retinal capillary obstruction and finally local ischemia (48). IR could lead to elevated levels of oxidative stress, increased malondialdehyde (MDA), a product of oxidative stress, and decreased antioxidants such as superoxide dismutase (SOD) and glutathione S-transferase (GSH-ST) (49). There was a close relationship between oxidative stress and endothelial cell dysfunction, which can cause diabetic microangiopathy (50). IR not only could diminish endothelial nitric oxide synthase (eNOS) activity, causing endothelial dysfunction, but also reduce nitric oxide production, which in turn leads to vasodilatory-contractile dysregulation, ultimately resulting in microcirculatory disorders and retinal damage (51–54). Mitochondrial dysfunction was strongly associated with IR and played an important role in the pathogenesis of DR (55). IR may speed up mitochondrial damage and thus promote apoptosis in retinal capillary cells (56, 57). In addition, some limited studies have shown that IR and DR may have a common genetic basis. For example, studies have found that DR was significantly associated with mutations in genes that express vascular endothelial growth factor (VEGF) (58). Additional studies have confirmed that blood VEGF levels were positively correlated with an index of IR (59). This suggested that expression of the VEGF gene may be one of the common genetic bases between IR and DR. Pro12Ala is located at the amino-terminus of the PPAR-γ 2 gene and was found to be associated with higher insulin sensitivity (59). Pro12Ala mutations may affect lipid metabolism, and pancreatic β-cell function, and be associated with the risk of IR (60). Meanwhile, it was also found that the alanine variant of Pro12Ala may be associated with a lower risk of DR (61). This suggested that Pro12Ala may be a common genetic base between IR and DR. Overall, these studies above imply that IR may mediate the mechanism of DR in type 2 diabetes. However, more studies are needed to prove these findings.

A high degree of heterogeneity was noted in our meta-analysis results, which may be due to the presence of many confounding variables. Heterogeneity decreased after the exclusion of Yao LT, et al’s study (a case-control study) and Pan Y, et al’s study (a cohort study), which may be related to the fact that the design type of the included studies was mainly cross-sectional. In addition, it was not excluded that the differences between studies in terms of study populations, number of participants, and adjusted confounders influenced heterogeneity. For example, this study involved populations from four different countries. The lowest number of participants was 208, while the highest was 4721. However, due to the small number of included studies, it was difficult for us to perform further subgroup analyses in this study. Nevertheless, the sensitivity analysis still presented a stable pooled result.

Despite obtaining a positive conclusion, our study still had the following limitations that merit further deliberation. First, the studies included in the analysis were mainly observational studies, with a predominance of cross-sectional studies. Their level of evidence was lower than randomized controlled trials or cohort studies Moreover, these studies were mostly concerned with the analysis of prevalence and lacked investigation of incidence. Second, it was worth considering that the number of studies included was very limited. This meant that the results of the study may not necessarily be applicable to a wider population. Additionally, there was a high degree of heterogeneity in these studies, and more studies were needed to ascertain whether research type, country, sample size, gender, and other study characteristics influenced the results of the analyses. Third, there were many confounding variables affecting the relationship between the TyG index and DR risk. The variables adjusted by different studies were not identical, which may have had an impact on the results. Finally, results based on observational studies could not show a causal relationship between the TyG index and DR risk. Therefore, it is essential to perform more high-quality cohort studies and basic research to obtain more reliable evidence.

## Conclusions

In conclusion, the existing evidence based on observational studies suggested that a higher TyG index was a potential predictor of DR risk in patients with type 2 diabetes. Considering the ease of obtaining the TyG index, more cohort studies are needed in the future to further identify the independent predictive role of the TyG index in DR incidence and prevalence, and it may also be compared with other DR risk prediction tools.

## Author contributions

JZ: Conceptualization, Data curation, Formal analysis, Investigation, Methodology, Software, Writing – original draft, Writing – review & editing. LZ: Data curation, Formal analysis, Writing – review & editing. YL: Resources, Supervision, Writing – review & editing.
