# Supplementary material for: Association between the triglyceride glucose index and diabetic retinopathy in type 2 diabetes: a meta-analysis
Source: Front Endocrinol (Lausanne). 2023 Dec 7;14:1302127. doi: 10.3389/fendo.2023.1302127 (PMC10733479; doi:10.3389/fendo.2023.1302127)
Supplement: Supplementary file 2 [file Table_2.docx]

**The search strategy of the PubMed database**

| Number | Keywords of the Research |
| --- | --- |
| #1 | Diabetic retinopathy [mh] |
| #2 | Diabetic retinopathy [tiab] OR Diabetic Retinopathies [tiab] OR diabetes retinopathy [tiab] OR retinopathy, diabetic [tiab] |
| #3 | #1 OR #2 |
| #4 | Triglyceride glucose index [tiab] OR TyG index [tiab] OR triglyceride and glucose index [tiab] OR triglyceride–glucose index [tiab] OR triglyceride/glucose index [tiab] OR Triacylglycerol glucose index [tiab] |
| #5 | #3 AND #4 |

Note: mh: MeSH; tiab: tittle/abstract

**The search strategy of the Embase database**

| **Number** | **Keywords of the Research** |
| --- | --- |
| **#1** | 'Diabetic retinopathy'/exp OR 'diabetic retinopathy' OR (diabetic AND retinopathies) OR (diabetes AND retinopathy) |
| **#2** | 'Triglyceride glucose index'/exp OR 'triglyceride glucose index' OR (('triglyceride'/exp OR triglyceride) AND ('glucose'/exp OR glucose) AND ('index'/exp OR index)) OR (triacylglycerol AND glucose AND index) OR (tyg AND index) |
| **#3** | #1 AND #2 |

Note: exp: exploded

**The search strategy of the Web of Science**

| **Number** | **Keywords of the Research** |
| --- | --- |
| **#1** | ((TS= (triglyceride glucose index)) OR TS= (TyG index)) OR TS= (Triacylglycerol glucose index) |
| **#2** | ((TS= (Diabetic retinopathy)) OR TS= (Diabetic Retinopathies)) OR TS= (diabetes retinopathy) |
| **#3** | #1 AND #2 |

Note: TS: topic

**The search strategy of the CNKI**

| **Number** | **Keywords of the Research** |
| --- | --- |
| **#1** | 主题=糖尿病视网膜病变 |
| **#2** | (主题=甘油三酯葡萄糖指数) OR (主题=TyG指数) |
| **#3** | #1 AND #2 |

Note: 主题: topic; 糖尿病视网膜病变: diabetic retinopathy; 甘油三酯葡萄糖指数: triglyceride glucose index
